# *Vibrio parahaemolyticus* Induced Necrotizing Fasciitis: An Atypical Organism Causing an Unusual Presentation

**DOI:** 10.1155/2013/216854

**Published:** 2013-12-17

**Authors:** Asim Ahmad, Lisa Brumble, Michael Maniaci

**Affiliations:** ^1^Community Internal Medicine, Mayo Clinic, Jacksonville, FL 32224, USA; ^2^Department of Infectious Disease, Mayo Clinic, Jacksonville, FL 32224, USA; ^3^Hospital Internal Medicine, Mayo Clinic, Jacksonville, FL 32224, USA

## Abstract

Background necrotizing fasciitis (NF) represents a life-threatening bacterial infection characterized by a rapid necrosis of deep subcutaneous tissue and facia underlying the skin. Despite its lethal nature, NF occurs infrequently, leaving many physicians unfamiliar with the disease process, common pathogens, and treatment strategies. Here we present a case of NF caused by an unlikely organism, *Vibrio parahaemolyticus*. We highlight the innocuous nature of initial presentation and the potentially devastating sequela.

## 1. Introduction

Necrotizing fasciitis (NF) represents a life-threatening bacterial infection characterized by rapid necrosis of deep subcutaneous tissue and fascia underlying the skin. Because of the very high mortality rate (80%) due to sepsis induced multiorgan systems failure, NF is considered both a medical emergency and a surgical emergency [1]. Despite its lethal nature, NF occurs infrequently, leaving many physicians unfamiliar with the disease process, common pathogens, and treatment strategies.

*Vibrio* species have been implicated in cases of NF associated with exposure to either seawater or shellfish. The causative organism in the vast majority of these cases is *Vibrio vulnificus*. Here we present a case of NF caused by an unlikely organism, *Vibrio parahaemolyticus*. We highlight the innocuous nature of initial presentation and the potentially devastating sequela. We then discuss the pathophysiology of NF, diagnosis, and treatment with emphasis placed on NF secondary to *Vibrio parahaemolyticus*.

## 2. Case Report

A 45-year-old lady presented to the emergency department with a chief complaint of severe left leg pain. Her past medical history was significant for modestly controlled diabetes mellitus type 2, morbid obesity with a BMI of 56, and chronic Hepatitis C infection. She had awoken earlier that morning experiencing subjective fevers, chills, and rigors. In addition, her left leg was so painful that she could not walk. She noted that she had been jet-skiing the previous day in brackish water and had sustained a small laceration on her left posterior calf.

Upon awakening, the leg was examined by patient's mother who reported minimal inflammation surrounding the area of injury. Because she could not walk, an ambulance was called and the patient was taken to a local emergency department (ED). She was triaged as having cellulitis and it was determined that she did not need to be seen emergently. While still in the ED waiting room, the patient's pain rapidly increased and she developed nausea and lightheadedness. As per her mother, the patient's ED triage vitals showed that she was afebrile and her blood pressure was within normal limits. Because of incessant pain and long wait, the patient was transferred directly by her mother to a different ED at a tertiary medical center.

On arrival, the patient's mother noted increased swelling over the left lower extremity. Physical exam performed in the ED revealed a woman in modest distress. She was afebrile but tachycardic with a heart rate of 110 beats/minute. Bilateral lower extremity examination revealed limb symmetry with chronic venostasis. A small abrasion over the left calf with associated erythema and tenderness was noted. This area was outlined with a skin marker. Of note, no vesicles, bullae, petechiae, or purpura was initially noted. There was no evidence of an ankle effusion or any ascending lymphangitis. Two peripheral blood cultures as well as a wound culture were obtained. Given concern for a salt water infection, doxycycline and ceftazidime were administered. Cefazolin was added to cover *Streptococcus*. Initial laboratory studies revealed the patient to be leukopenic with a WBC of 2400 (neutrophil predominate) and thrombocytopenic with a platelet count of 94 K. Three-view film of the left tibia/fibula demonstrated a soft tissue abnormality in the mid-medial left lower extremity; however, emphysema was not seen on any view.

The patient was admitted to the Internal Medicine service. Upon reexamination within one hour of admission, the left leg was noted to be 3 times the size of the right. Furthermore, the left leg had become discolored, described as “very dark” from below the knee to the distal aspect of the foot. Numerous large bullae were now beginning to form. The area of involvement had spread well beyond the area initially demarcated by the skin marker. Aspiration of a bullae yielded serous fluid which was sent for gram statin and culture. Laboratory data at this time revealed a hemoglobin drop from 13.2 g/dL to 11.7 g/dL. The white blood cell count was now 15.4 K with 51% neutrophils. The prothrombin time (PT) was elevated at 32 seconds and the activated partial thromboplastin time (APTT) was elevated at 58 seconds. A quantitative D-dimer was positive at 18.4 *μ*g/mL, and a fibrin monomer detection was positive, likely indicating that the patient was in a state of disseminated intravascular coagulation (DIC). Lactic acid level was measured at 19 mmol/l. Serum creatinine had increased from 1.0 to 3.3 mg/dL, and creatinine phosphokinase (CPK) was 11,500 *μ*/L. Liver enzyme testing revealed an alanine aminotransferase (ALT) of 144 *μ*/L, an aspartate aminotransferase (AST) of 270 *μ*/L, and an alkaline phosphatase of 58 *μ*/L. Blood cultures, 2 out of 2, were positive for gram-negative bacilli.

These findings were concerning NF of the left leg with distributive septic shock and multiorgan dysfunction. The Infectious Disease team was consulted for antibiotic recommendations. Therapy was modified from the original ED treatment plan and consisted of broad-spectrum therapy with cefepime, levofloxacin, doxycycline, and clindamycin. The patient's DIC was treated with fresh frozen plasma and cryoprecipitate. The patient was also aggressively volume resuscitated with lactated ringer solution given concern for the acute renal failure, shock liver, and metabolic acidosis. General surgery was immediately consulted for fasciotomy. Before the patient could undergo emergent surgery, she experienced an asystolic cardiac arrest. Cardiopulmonary resuscitation (CPR) was performed and the patient was stabilized. Lifesaving measures included intubation for respiratory failure and blood pressure support with epinephrine, atropine, norepinephrine, and vasopressin. An arterial blood gas done at that time demonstrated a severe metabolic acidosis with a pH of 6.9. This was treated with multiple doses of bicarbonate solution and initiation of continuous venovenous hemofiltration (CVVH).

Once the patient was stabilized, she was taken to the operating room for emergent fasciotomy. Despite this intervention, the patient's condition remained critical and her vital signs repeatedly became unstable. Later that morning, she again went into asystolic cardiac arrest. Despite maximum heroic efforts, she was unable to be resuscitated. Later on that day, both blood cultures and cultures from the leg grew *Vibrio parahaemolyticus*. These samples were sent to Mayo Medical Laboratories in Rochester, MN, USA, and DNA sequencing confirmed that the organism was *Vibrio parahaemolyticus*.

## 3. Discussion

Necrotizing fasciitis may be localized to any area of the body though common site includes the perineum, abdominal wall, and the extremities. It is often the result of disruption of the skin (laceration, insect bite, surgical incision, injection, burn, etc.) [2]. Once the skin is penetrated by microbes, the release of chemical mediators results in pyrogenic endotoxins and exotoxins. Continued release of these cytokines eventually leads to damage of the endothelial cells, resulting in inflammation, edema, and decreased blood flow resulting in subsequent necrosis [3]. Furthermore, microbes such as the *Vibrio* species can secrete an extracellular toxin which can lead to soft-tissue damage. Because the fascial layers beneath the skin are not well supplied by blood vessels, a further decrease in blood flow leads to an inhibition of the inflammatory process used to fight infection and disrupts the delivery of antibiotics to the affected area. This is the reason why rapid surgical excision and debridement of the affected tissue are necessary to reduce bacterial load and stop further spread of infection.

Necrotizing fasciitis usually manifests rather nonspecifically with symptoms such as fever, chills, malaise, and aches. The skin generally becomes warm, tender, and edematous with localized pain described as “out of proportion to physical exam” [4]. Unfortunately, these symptoms are often overlooked upon initial evaluation and blamed on entities such as viral infections, muscle strain or, as in this case, on cellulitis. Laboratory data associated with NF are also rather nonspecific and include leukocytosis, hyperglycemia, increased creatinine, new onset coagulopathy, and metabolic acidosis [5]. The hallmark radiographic finding of soft tissue air on plain radiograph is seen in about 57% of patients [6]. Physical exam can reveal crepitus over the affected area 37% of the time [6]. Predisposing conditions making one prone to NF include diabetes mellitus, liver disease, obesity, smoking, peripheral vascular disease, alcohol abuse, and intravenous drug use.

In terms of etiology, NF can be divided into three types. Type I refers to a polymicrobial infection comprised of aerobic gram-negative bacteria and anaerobic gram-negative and gram-positive bacteria. Patients with diabetes mellitus account for a subset of this group. Type II is the most common type and consists of Group A hemolytic *Streptococcus* sometimes in conjunction with *Staphylococcus*. Interestingly, in 50% of these infections, an obvious portal of entry is not identified [7]. Finally, Type III NF is caused by marine *Vibrio* gram-negative rods. It is often caused by a puncture wound from a fish or an insect bite or a cut that has been exposed to seawater or shellfish. The clinical course of these infections is more rapid than in cases of either Type I or Type II NF although they tend to occur more infrequently.

This patient suffered from DNA sequencing proven *Vibrio parahaemolyticus* NF. The *Vibrio* species are short, oxidase-positive, and gram-negative bacilli that are often curved and actively motile. They are free living bacteria often isolated from fish and shellfish [8]. There has been a rapid increase in the incidence of infections caused by *Vibrio parahaemolyticus* since the mid-1990s. This increase is associated with a new clonal group and may be related to rising water temperatures in shellfish growing areas, creating a more favorable growth environment [9].

Wound infections due to *Vibrio parahaemolyticus *associated with exposure to brackish water are typically mild. However, mortality rates approaching 3% are reported [10]. Clinically, it commonly presents as either gastroenteritis or primary septicemia associated with a wound injury. Wound infection generally occurs about one day after injury and manifests as swelling, pain, ecchymosis, and blistering. More aggressive infections may present as hemorrhagic bullae, necrosis, gangrene, and/or subcutaneous bleeding. The septicemia associated with this often leads to multiple-organ failure and death [11]. Patients with liver disease and diabetes mellitus have a higher risk of developing sepsis. Chuang et al. reported an underlying chronic illness being present in 55% of patients with primary *Vibrio* wound infections and in 94.7% of patients with primary *Vibrio* septicemia [12]. This is likely secondary to an immunocompromised state associated with these disease states. Also, with liver disease, an iron overload state can occur which provides a more favorable environment for *Vibrio* growth since the organism is impaired in its ability to extract iron from transferrin [13]. Incidences of wound infections are more common in Florida during the months of April to October secondary to warmer water temperatures.

As alluded to earlier, the primary treatment for NF is prompt surgical intervention with debridement in order to obtain source control. This often requires multiple surgeries. Faucher et al. report that, in a case series of 57 patients with NF, an average of 4.1 surgeries was undertaken [14]. Despite rapid intervention, mortality may still be quite high. For instance, Howard et al. reported that, in 24 patients with NF caused by the *Vibrio* species requiring surgical debridement, 11 died [15]. In conjunction with surgical debridement, antibiotics should also be administered. For mild *Vibrio* wound infections without signs of NF, a tetracycline or a fluoroquinolone is likely sufficient treatment. Duration of therapy is guided by response and is generally 5–7 days. In patients with septicemia, risk factors for septicemia, or NF, a more aggressive antimicrobial therapy in an ICU setting is often required. Antibiotic therapy in these cases often involves a tetracycline in combination with a third generation cephalosporin, an aminoglycoside, or chloramphenicol [16]. Duration is once again based on clinical response in addition to degree of immunosuppression but generally lasts two or more weeks.

Necrotizing fasciitis represents both a medical emergency and a surgical emergency. Prompt diagnosis and treatment are paramount. Despite the devastating consequences, NF occurs infrequently enough so that most providers may not consider it. This is further confounded by the relatively benign initial presentation of NF. Through this case report we hope to have highlighted possible presenting characteristics, the importance of serial physical exams, and how laboratory data as well as patients comorbidities can be used to create an optimum treatment plan. We also have highlighted an unusual cause of NF, *Vibrio parahaemolyticus*. One must obtain a thorough history and have a high index of suspicion to make this diagnosis.
